# Adherence to a Standardized Chemotherapy Order form for Colorectal Cancer in a Referral Teaching Hospital, Mashhad, Iran

**Published:** 2019

**Authors:** Mohamad-Mehdi Kooshyar, Anoosheh Maruzi, Azar Fani Pakdel, Sepideh Elyasi, Ali Taghizadeh-Kermani, Mahdi Akbarzadeh, Seyed Amir Aledavood

**Affiliations:** a ***Department of Hematology and Oncology, Faculty of Medicine, Mashhad University of Medical Sciences, Mashhad, Iran. ***; b ***Department of Clinical Pharmacy, Faculty of Pharmacy, Mashhad University of Medical Sciences, Mashhad, Iran. ***; c ***Cancer Research Center, Mashhad University of Medical Sciences, Mashhad, Iran.***

**Keywords:** Colorectal cancer, Chemotherapy, Medication error, Standard form, Teaching hospital

## Abstract

Using standardized forms for prescription and administration of medications is one of the main solutions for reducing medication errors in the chemotherapy process. Considering the high prevalence and mortality rate of colorectal cancer, in this study we tried to design and validate a standard printed form and evaluate oncologists’ and nurses’ adherence to this form. This cross-sectional study was performed in Omid hospital, Mashhad, Iran from January 2015 to October 2015. A Chemotherapy form including various demographic and clinical parameters and approved chemotherapy regimens for colorectal cancer was designed by the clinical pharmacist and validated by clinical oncologists working in this center. All eligible patients admitted in this center during this period of time were included in the study. Adherence of the oncologists and nurses to this form and probable medication errors were identified by the pharmacy student. Sixty-seven patients with colorectal cancer and a total of 251 chemotherapy courses were evaluated. All patients received regimens compatible with developed form but in 206 courses (98.56%) of chemotherapy dosing error happened and in most of cases patients received lower than calculated dose (37.8%). Three errors occurred in administration step by nurses which they infused the medication in shorter than recommended duration. In general, oncologists’ adherence with developed form for chemotherapy of colorectal cancer was relatively high, except in dose calculation. Avoiding from rounding the calculated medications’ doses and precise calculation of patients’ body surface area can prevent most of medication errors and reduce risk of adverse drug reaction occurrence.

## Introduction

One of the most serious challenges in medical systems all around the world is Medical errors. Medication errors are one of the most prevalent medical errors (1). Institute of medicine of the United States has classified medication errors among the top five medical errors which can lead to serious injury and even Death (2). The standard definition for medication errors is suggested by The National Coordinating Council for Medication Error Reporting and Prevention (NCC MERP) which is “any preventable event that may cause or lead to inappropriate medication use or patient harm while the medication is in the control of the health care professional, patient, or consumer”. In 2000 an annual report stated that 48-98 thousands of deaths due to drug adverse reactions and complications have occurred in the United States that seven thousands of these cases are related to medication errors (3). 

In Iran, no official statistics are available regarding the rate of medication errors, but it is estimated to be too high (4). 

Medication errors have many negative impacts on patients, nurses, and health organizations leading to the decreased quality of care. They are not only significantly effective on patients’ outcome but also on their length of hospital stay and treatment cost. Thus, identifying the causes and taking to account some measures for reduction of medication errors can be considered a priority (5-8). 

Colorectal cancer is the most common gastrointestinal cancer and the third leading cause of cancer death after lung and breast cancers. Moreover, it is the fourth most common cancer in Iran (9). 

Antineoplastic drugs are more prone to medication errors, due to narrow therapeutic index which can lead to toxicity occurrence even at therapeutic doses, complexity of chemotherapy regimens, higher sensitivity of cancer patients to drug adverse reaction and their several comorbidities (10). Despite many attempts to enhance safe administration of antineoplastic drugs, there are lots of reports about medication errors (11, 12). According to statistics in 2010 , 21% of medication errors in chemotherapy are fatal and 23% of them causes permanent disability (13).To enhance the safety of chemotherapy administration, all members involved in the process of chemotherapy should play a role in standardization of chemotherapy protocols (14,15).

Numerous studies have shown that standardized chemotherapy forms can improve oncology patients’ care and reduce errors (16-20). The standard form is a written order form including different variables and prescription parameters such as diagnosis, height, weight, body surface area, start date and time, dosage (e.g., mg/m^2^), dose (mg), solution diluent (drips only) and volume (drips only), infusion rate (drips only), route (i.e., IV push or IV drip), frequency of administration, and total number of scheduled dose. (21). 

However, standard forms have limited use in Iran oncology wards. So, in this study we aimed to design and validate a standard form for treatment of colorectal cancer in an oncology teaching hospital and evaluate treatment team adherence to this form with a focus on medication errors occurrence. 

## Experimental

This was a cross-sectional study conducted during January 2015 to October 2015, at an oncology medical center, Omid Hospital affiliated to Mashhad University of Medical Science, Iran.

Patients with colorectal cancer diagnosis receiving chemotherapy regimens whose ages are between 18 and 70 years are included in this study. Patients with baseline hepatic (serum transaminase levels > 2-3 times higher than the upper limit of normal), renal (estimated glomerular filtration rate < 50 mL/min) or heart (ejection fraction < 50, if echocardiography was available) failure were excluded, as they need specific dose adjustment for some chemotherapeutic agents.

The study protocol was approved by the local Ethics Committee of Mashhad University of Medical Sciences (930637). All participants signed written consent forms.

**Table 1:** **classification of patients regarding their cancer anatomical stage**

**Frequency (%)**	**Stage**
**30.2**	**IIA**
**5.2**	**IIB**
**9.5**	**IIC**
**1.7**	**IIIA**
**37.1**	**IIIB**
**9.5**	**IVA**
**6.9**	**IVB**

**Table 2:** **Frequency of prescribed chemotherapy regimens**

**Regimen** *	**Frequency [number] (percentage [%])**
**FOLFIRI**	** 31 (12.4)**
**FOLFIRI+bevacizumab**	**8 (3.2)**
**FOLFOX IV**	** 173 (68.9)**
**FOLFOX VI**	**3 (1.2)**
**FOLFIRI+cetuximab**	**12 (4.8)**
**FOLFOXFIRI**	**1(0.4)**
**FOLFOX IV+cetuximab**	**10 (4)**
**mFOLFOX VI**	**11(4.4)**
**mFOLFOX VI+cetuximab**	**2 (0.8)**

* **The details of each regimen are available in supplement**

**Table 3:** **dose calculation error percentage for different chemotherapeutic agents**

**Name of drug**	**Number of courses the drug used in**	**Range of error (%)**	**Mean of error** 1 **(%)**	**Std. Deviation**
**folinic acid**	**251**	**0-16**	**3.06**	**3.755**
**5-FU blous**	**250**	**0-17**	**3.70**	**4.331**
**5-FU inf** *	**249**	**0-32**	**3.65**	**5.662**
**oxaliplatin**	**199**	**0-26**	**4.77**	**5.277**
**irinotecan**	**57**	**0-21**	**4.54**	**4.018**
**bevacizumab**	**6**	**0-9**	**4.00**	**4.099**
**cetuximab**	**24**	**0-11**	**5.08**	**2.873**

* **5-Fluorouracil infusion**

1 **The absolute mean of error has been reported for errors higher than 5% (regardless of its positivity or negativity).**

**Table 4:** **Distribution of dose calculation error in colorectal cancer patients**

**dosing**	**Frequency (%)**
**Suitable**	**27 (10.8)**
**Overdose**	**58 (23.1)**
**Underdose**	**95 (37.8)**
**over/under** 1	**71 (28.3)**

1 **some medication in regimen have been prescribed with lower than calculated doses and some of them with higher than required dose.**

**Table 5:** **Frequency of different types of medication errors based on psychological approach**

**Type of medication error**	** N (%)**	
**Mistakes**	**Knowledge-based errors**	**-**	
	**Rule-based errors**	**206 (98.5)**	
**Skill-based errors**	**Action-based errors**	**3 (1.5)**	
	**Memory-based errors**	**-**	

**Table 6:** **comparison of mean percentage error between different chemotherapy regimens**

**Regimen (I)**	**Regimen (J)**	**Mean Difference** **in error (I-J)**	**Std. Error**	**Sig.** *	**95% Confidence Interval**	
					**Lower Bound**	**Lower Bound**
**FOLFIRI**	**FOLFIRI+bevacizumab**	**.63333**	**1.21936**	**.604**	**-1.7693**	**3.0359**
**FOLFIRI**	**FOLFOX IV**	**2.12466** *	**.60605**	**.001** *	**.9305**	**3.3188**
**FOLFIRI**	**FOLFOX VI**	**-.61667**	**1.85559**	**.740**	**-4.2729**	**3.0395**
**FOLFIRI**	**FOLFOX IV+cetuximab**	**4.07333** *	**1.11896**	**.000** *	**1.8686**	**6.2781**
**FOLFIRI**	**mFOLFOX VI**	**-.02576**	**1.08014**	**.981**	**-2.1540**	**2.1025**
**FOLFIRI+bevacizumab**	**FOLFOX IV**	**1.49133**	**1.10820**	**.180**	**-.6922**	**3.6749**
**FOLFIRI+bevacizumab**	**FOLFOX VI**	**-1.25000**	**2.07461**	**.547**	**-5.3378**	**2.8378**
**FOLFIRI+bevacizumab**	**FOLFOX IV+cetuximab**	**3.44000** *	**1.45358**	**.019** *	**.5759**	**6.3041**
**FOLFIRI+bevacizumab**	**mFOLFOX VI**	**-.65909**	**1.42391**	**.644**	**-3.4647**	**2.1465**
**FOLFOX IV**	**FOLFOX VI**	**-2.74133**	**1.78451**	**.126**	**-6.2575**	**.7748**
**FOLFOX IV**	**FOLFOX IV+cetuximab**	**1.94867**	**.99666**	**.052**	**-.0151**	**3.9125**
**FOLFOX IV**	**mFOLFOX VI**	**-2.15042** *	**.95288**	**.025** *	**-4.0279**	**-.2729**
**FOLFOX VI**	**FOLFOX IV+cetuximab**	**4.69000** *	**2.01724**	**.021** *	**.7153**	**8.6647**
**FOLFOX VI**	**mFOLFOX VI**	**.59091**	**1.99597**	**.767**	**-3.3419**	**4.5237**
**FOLFOX IV+cetuximab**	**mFOLFOX VI**	**-4.09909** *	**1.33894**	**.002** *	**-6.7373**	**-1.4609**

* **One-way ANOVA multiple comparison test.**

**Table 7:** **Epidemiology of medication error in chemotherapy: Comparison with published literatures**

**present study**	**Walsh ** ***et al *** **(25)**	**Dhamij ** ***et al*** **(26)**	**Rinke ** ***et al*** **(34)**	**Fyhr ** ***et al*** **(33)**	
**2015**	**2008**	**2012**	**1999-2004**	**1996-2008**	**Year of study**
**8 months**	**9 months**	**8 months**	**5 years**	**12 years**	**Length of the study**
**Cross-sectional**	**Retrospective**	**Retrospective**	**Retrospective**	**Retrospective**	**Design**
**18-70**	**All ages**	**<18**	**<18**	**All ages**	**Patients age (y)**
**62**	**8.1**	**11**	**1**	**NS**	**ME rate (%)**
**98**	**36**	**13**	**10**	**42**	**Errors in ordering (%)**
**2**	**56**	**43**	**48**	**16**	**Errors in administering (%)**
**57.8**	**NS**	**9**	**22.9**	**45%**	**Dosing error rate (%)**
**1.2**	**NS**	**26**	**NS**	**NS**	**Rate of error in duration of infusion**
**No**	**NS**	**NS**	**12.2**	**NS**	**Wrong route**
**No**	**NS**	**NS**	**NS**	**0.08**	**Wrong patients**
**No**	**NS**	**NS**	**NS**	**30**	**Wrong drug**

**ME: medication error.**

A standardization form was prepared by the clinical pharmacist based on international available guidelines (22, 23) and reviewed and approved by all clinical oncologists working in these centers. Patients’ demographic and clinical data including age, weight, height, body surface area (BSA), past medical and drug history, diagnosis and stage of disease were registered in this form. Moreover, all of the approved chemotherapy protocols for colorectal cancer mentioned in various guidelines were collected in this form and patients’ regimen (including dose of drugs, root of administration and duration of the treatment course) and also drugs’ brand were specified in it. In addition, acute adverse drug reactions which occurred for patient during drug administration were also recorded (The standard form was presented in supplement).

The form was completed by the clinical oncologist at time of patients’ reference to oncology department for admission. These forms were collected by the pharmacy student in oncology wards. All medication errors (e.g. selection of regimen, dose of medication, root of administration, duration of therapy, selection of suitable diluent, rate of infusion) and possible adverse effect were evaluated and recorded by pharmacy student. Furthermore, after data collection, for each chemotherapy session, BSA was re-calculated and compared with the BSA calculated by the oncologist. BSA calculation was based on Du Bois formula, according to its high reliability and accuracy among all available formulas for its calculation, using patients’ actual body weight (24). It should be mentioned that this formula was also routinely used by the oncologists of our center for BSA calculation. 

For evaluation of the medication dose, if prescribed dose was more than 5% over or under the calculated dose, it was considered as a medication error based on previous studies’ recommendation (25, 26).

Results have been shown as mean ± standard deviation (SD) or number (percentages) for nominal variables. Kolmogorov-Smirnov test was used to assess the normality of the variables distributions. Data analysis was performed using the SPSS 16.0 statistical package. Univariate associations were assessed using the Chi-square test. One-Way (ANOVA) and Kruskal-wallis were respectively used for comparison of data with normal and non-normal distribution in different groups. Statistical significance was set at *P *< 0.05. 

## Results

*Population data*

The study population consisted of 67 patients, 36 male (53.7%), and 31 female (46.3%). The mean age of population was 52.73 ± 12.19 years. The average weight and BSA of patients were 61.084 ± 12.9 kg and 1.81 ± 1.64 kg/m^2^ respectively. Regarding anatomical stage of the cancer, most of patients were in stage IIIB (37.1%) and IIA (30.2%) (Table 1). The majority of patients (85.6%) had metastatic colorectal cancer. FOLFOX IV was the most common prescribed chemotherapy regimen during the study (Table 2).

*Adherence to standardized*

*chemotherapy protocol: medication errors*

During these 8 months, 251 sessions of chemotherapy were evaluated with an average of 4.88 ± 3.26 sessions for each patient. During these 251 sessions of chemotherapy, 209 medication errors were recorded. These errors had occurred in treatment process of 53 patients (79.1%). Most of them (98.56%) happened in prescription step by the physicians and 3 errors occurred by nurses during administration step. In all sessions, prescribed chemotherapy regimen was compatible with approved regimens mentioned in standard form. But in 209 treatment courses (83.26%) dose calculation error happened which included 57.63% of prescribed drugs. Most of dose calculation errors (83.33%) and the highest mean percentage of error (5.08 ± 2.87%) were related to cetuximab (Table 3). In most of these treatment courses patients received lower than recommended doses (37.8%) (Table 4). 

The most prevalent reason for dose calculation error was miscalculation of BSA which happened in 25.5% of chemotherapy sessions and for 22.39% of patients. The mean percentage of error was 8.03 ± 3.99%. BSA was considered lower than actual value in most of cases (52.5%). Actually in most of these patients BSA was miscalculated from the beginning of the chemotherapy, but in 8 courses of chemotherapy (3%), using the baseline BSA in the next courses of chemotherapy was the cause of the error, as patients’ weight had changed during the treatment course. Three errors which are found in the administration of chemotherapy were related to the time of infusion. In two cases folinic acid infusion time for patients was less than recommended time (2 h) in FOLFIRI and FOLFOX IV regimens. In the third case oxaliplatin should be infused in 2 h which was administered in about 1 h. 

Selection of carrier solution (type and volume) and root of drug administration were correct in all patients.

The classification of medication errors based on a psychological approach which is usually preferred method for classification of medication errors, as it explains events rather than merely describing them (27) is summarized in Table 5. The rule-based errors were the most common type (98.5%). The mean percentage of dosing errors was compared between different chemotherapy regimens for colorectal cancer which were significantly different between some of regimens (*P *value < 0.001) (Table 6).

## Discussion

Colorectal cancer is the third leading cause of cancer death in the world (23, 28). Because of the narrow therapeutic index of chemotherapy agents and their high toxicity, medication errors in chemotherapy are of particular importance. It is reported that millions of deaths due to preventable medication errors occur annually in cancer patients. Therefore, prevention of medication errors in this population should be a priority (29, 30).

Development and standardization of therapeutic guidelines and protocols in format of a standardized printed or electronic form is one of the main methods for medication error reduction particularly in chemotherapy. It also can improve physician prescribing patterns and prescription completeness (19, 31). There are some studies performed in oncology ward of hospitals evaluating medication errors occurrence after implementation of a standardized printed/electronic prescription form. Dumasia *et al.* performed a study during 1999 to 2003 in oncology ward of a teaching hospital in USA to evaluate quality performance improvement with implementation of standard chemotherapy order forms. They reported that the average order completeness have improved from 45% to 81% when they designed a standard written form and replaced the unstandardized blank order sheets with it. Implementation of the electronic chemotherapy form increased the completeness to 93% (31). Another study was carried out by Voeffray and colleagues in Switzerland to assess the effect of a computerized physician order entry (CPOE) system on the number of errors in prescription. They declared that using CPOE system reduced errors in chemotherapy prescription from 15% to 5% (32). 

But to the best of our knowledge it is the first study performed particularly on colorectal cancer patients.

Moreover, in current study the standard form containing approved regimens for the treatment of various stages of colorectal cancer and the recommended dose for each medication, the duration of its administration and suitable carrier, was prepared which could reduce prescription writing time and errors. In our cross-sectional study a total of 251 sessions of chemotherapy and 1036 drugs were studied. Overall 209 medication errors were recorded that 206 errors (98.56%) had happened in the prescription step and 3 (1.44%) in administration step by nurses. 

In a study performed in Turkey on patients receiving chemotherapy regimens which was conducted in 18 chemotherapy departments in 2015, the most common reported errors were prescribing the wrong dose of medications by physicians (65.7 %) and receiving the improper drugs (50.5 %) (30). Our findings are compared with other previous studies in Table 7 (25, 26, 33, 34). The medication error rate was significantly higher in our study, and errors in prescription step were dominant, in contrast to previous studies which administration step errors were usually more common. The high rate of prescription error in this center may be due to limited access to medications and their high price which forced the oncologists in most of the cases to round the doses to the available dosage form to reduce the cost. But employing trained nurses in chemotherapy administration was effective for reduction of administration phase errors. 

The dose calculation errors have occurred in 83.26% of patients; sixty-eight percent of them have received less than the required dose. Receiving lower than therapeutic dose can reduce treatment efficacy and dose higher than the required dose put patients at risk of side effects (35). One of the main causes of dose calculation error is miscalculation of body surface area resulting from inaccurate measurements of patients’ height and weight and sometimes estimation of these data instead of precise calculation.

We also classified medication errors based on psychological approach which is the preferred method. This method distinguishes between errors in planning an act and errors in its execution. In this classification mistakes can be divided into (i) knowledge-based errors and (ii) rule-based errors and failures of skill into (iii) action-based errors (’slips’, including technical errors) and (iv) memory based errors (‘lapses’).Knowledge-based errors can be related to any type of knowledge, general, specific, or expert. For example it is a general knowledge that penicillin can cause allergic reactions; Ignorance of this fact could lead to a knowledge-based error. Rule-based errors are misapplication of a good rule or the failure to apply a good rule and the application of a bad rule. An action-based error is defined as the performance of an action that was not what was intended; For instance, a slip of the pen, when a doctor want to write diltiazem but writes diazepam or addition of wrong amount of a medication to an infusion bottle. Memory-based errors occur when something is forgotten; for example, giving penicillin, knowing the patient to be allergic (27).

In this study we found that most of errors were in rule-based error group (98.5%). This type of error can be prevented mostly by improving roles. Providing suitable and applicable standard forms based on available evidence based guidelines is one of possible action in this field. Other errors were action-based errors. Training can be effective in reducing this type of error especially for the nurses to signify the importance of administrating phase for chemotherapeutic agents to them.This study suffered some limitations. First, time of the study was short and thus limited number of patients was included. Second, despite of our initial planning to perform a multi-center study and validating the standard form in cooperation with oncologists of three hospitals in Mashhad, Iran, finally the form was used and evaluated in just one center. Third, as we did not perform a pre-implementation phase study, we could not compare pre and post-implementation error rate. Forth, due to the long duration of infusion for some of medication (up to 46 h) in some regimens the evaluation of infusion time may be troublesome in these cases as the pharmacy student was not resident in the ward and consequently in these cases she rely on nurses’ reports.

## Conclusion

In conclusion, in this oncology center the adherence of oncologists to standardized protocol is relatively high in term of correct regimen selection for treatment of colorectal cancer. But most of medication errors happened in dose calculation resulting in underdosing in most of cases which could cause treatment failure. So, avoidance from rounding the calculated medication doses and precise calculation of patients’ BSA before determination of medications doses can improve quality of chemotherapy and patients’ outcome and reduce adverse drug reaction occurrence.
